# Distress Houses

**Published:** 1859-03

**Authors:** 


### DISTRESS HOUSES.

M. Viennot, of Paris, proposes to erect ornamental columns throughout New York, below Fourteenth street, at his own expense, receiving pay therefor in the exclusive privilege of using the wall of those columns for placards for twenty years, after which they revert to the city. It is sufficient to say that these columns are to be used as urinals. If any plan could be devised to keep these places as neat as a new pin, as to sight and sense, they would be productive of great, very great good. The inconvenience, the discomfort, and the positive danger which result from the want of these facilities in a large city cannot be denied. As to the positive danger, and the daily injury, any experienced physician will give a prompt testimony. For want of the conveniences referred to, violations of public decency are of daily occurrence, while the prostitution of the corners, areas and recesses of private property for these purposes is shameful, and is an opprobrium to any civilized city.

